# Management of Bleeding due to Idiopathic Colonic Varices

**DOI:** 10.14309/crj.0000000000001791

**Published:** 2025-08-01

**Authors:** Abhimati Ravikulan, Richard Gearry, Jan Kubovy

**Affiliations:** 1 Department of Gastroenterology, Christchurch Hospital, Christchurch, New Zealand; 2 Canterbury District Health Board, Christchurch, New Zealand; 3 Otago University, Dunedin, New Zealand; 4 New Zealand Nutrition Foundation, Auckland, New Zealand; 5 Crohn's and Colitis, Lower Hutt, New Zealand

**Keywords:** Colonoscopy, idiopathic colonic varices, endoscopy, lower GI bleeding, endoscopic intervention

## Abstract

Lower gastrointestinal bleeding due to idiopathic colonic varices (ICV) is very rare. We present a 66-year-old man with recurrent hematochezia but without history of liver disease or portal hypertension. Colonoscopy revealed extensive varices throughout the colon. There was no biochemical or radiological evidence of cirrhosis or portal hypertension. The underlying etiology of ICV is unknown, and management of this condition is challenging without standardized treatment protocols. Nonselective beta-blockers could be considered despite the unknown ICV pathophysiology and their effect in this clinical entity. The successful management in our case included immediate endoscopic treatment and secondary prophylaxis with carvedilol.

## INTRODUCTION

Colonic varices (proximal to rectum) are a very rare cause of lower gastrointestinal bleeding. The occurrence of colonic varices in the absence of portal hypertension (with or without cirrhosis), termed idiopathic colonic varices (ICV), is rare.^1^ No consistent risk factors have been identified in the literature.

Symptomatic ICV often present with recurrent hematochezia and/or chronic anemia. The pathogenesis remains unknown, although some reports suggest congenital venous anomaly or familial predisposition.^2^

The management of ICV is challenging due to the lack of standardized treatment protocols. The published approaches vary from conservative management, endoscopic interventions to surgical resection in cases of severe or refractory bleeding. Nonselective beta-blockers (NSBBs) are the cornerstone of the management of portal hypertension-related esophageal varices^3^; however, little is known about their application in ICV.

## CASE REPORT

A 66-year-old White man investigated for iron deficiency anemia underwent elective gastroscopy and a colonoscopy. The gastroscopy was normal, without endoscopic evidence of portal hypertension. However, extensive varices were found throughout his entire colon. Subsequent abdominal computer tomography (CT) scan showed no evidence of cirrhosis, portal hypertension, splenomegaly, portal vein thrombosis, or mesenteric vein occlusion.

He had essential hypertension that was well managed with candesartan, felodipine, and metoprolol. There was no personal or family history of liver disease, bowel disease, venous thrombosis, or portal hypertension. He had no previous abdominal surgery. He worked as a truck driver, did not smoke, and had no insignificant alcohol intake.

**Figure 1.**
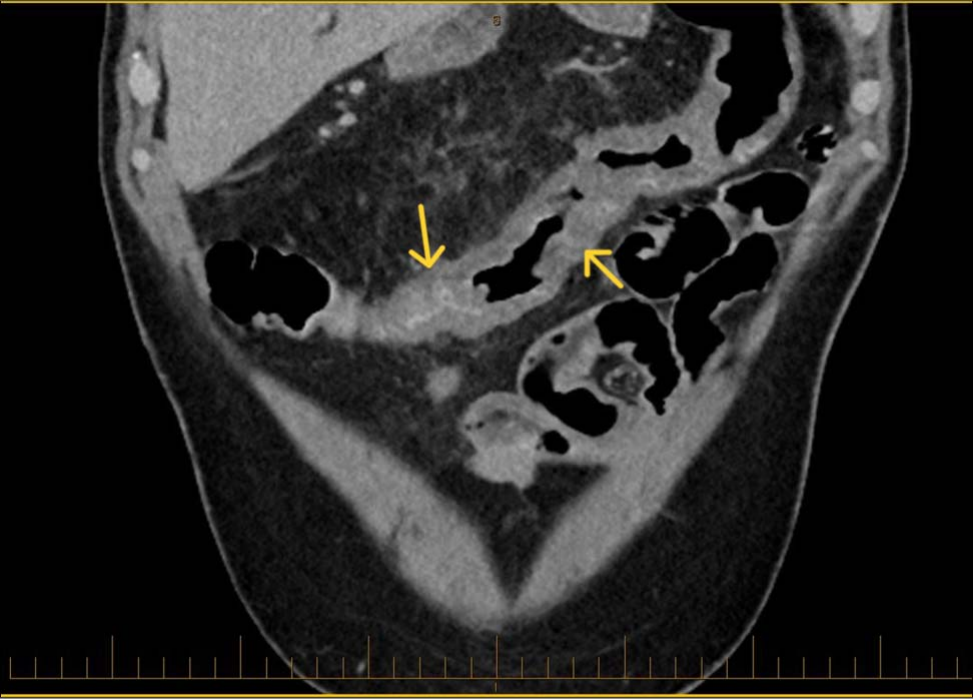
CT coronal view showing colitis. Arrows showing colon wall thickening/oedema likely reflecting varices.

**Figure 2.**
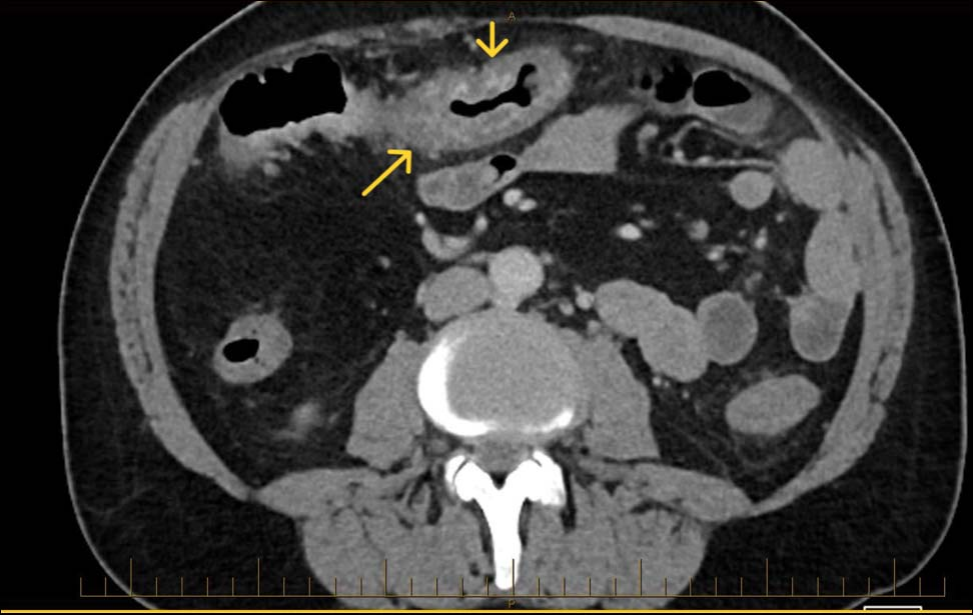
CT axial view showing colitis. Arrows showing colon wall thickening/oedema likely reflecting varices.

**Figure 3.**
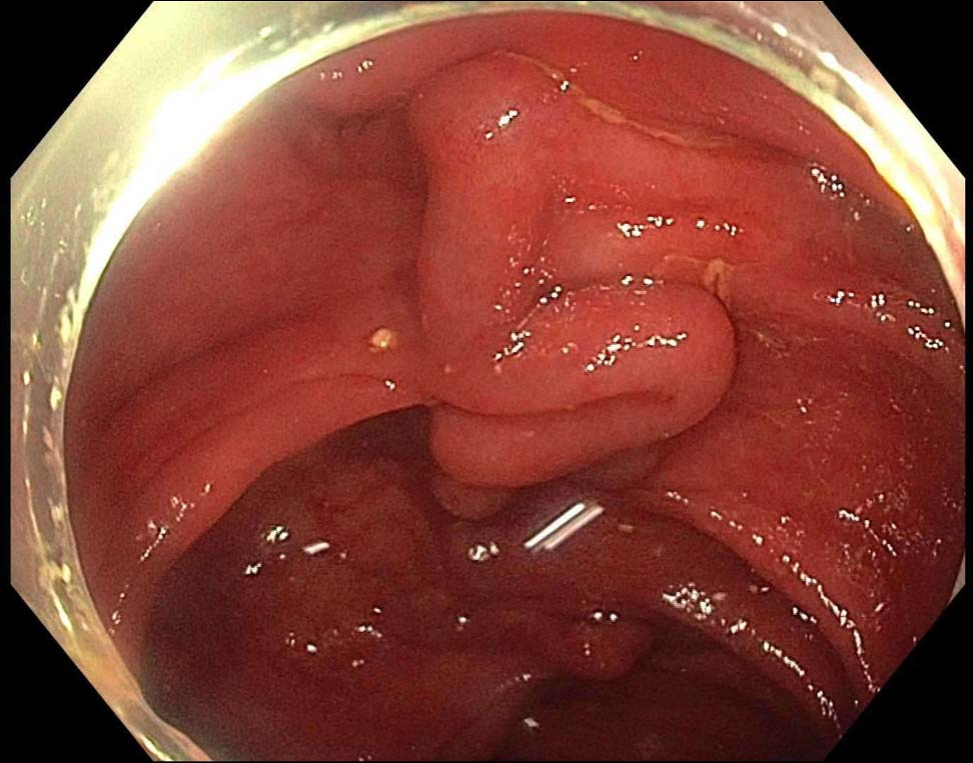
Colonic varices.

**Figure 4.**
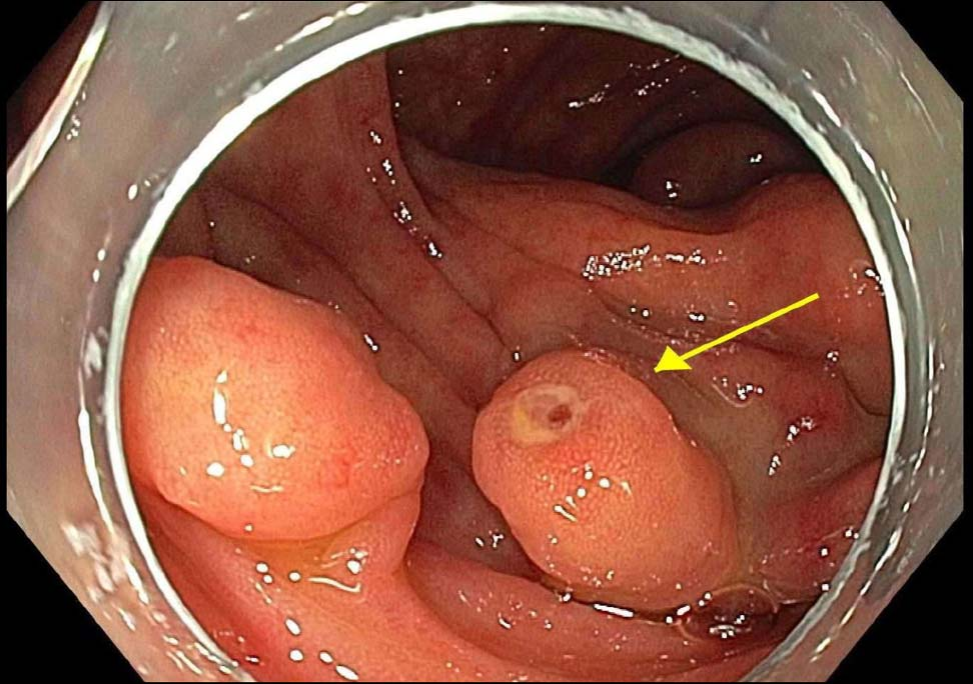
Culprit varix with stigmata of bleeding. Arrow showing cuprit varix with bleeding point.

**Figure 5.**
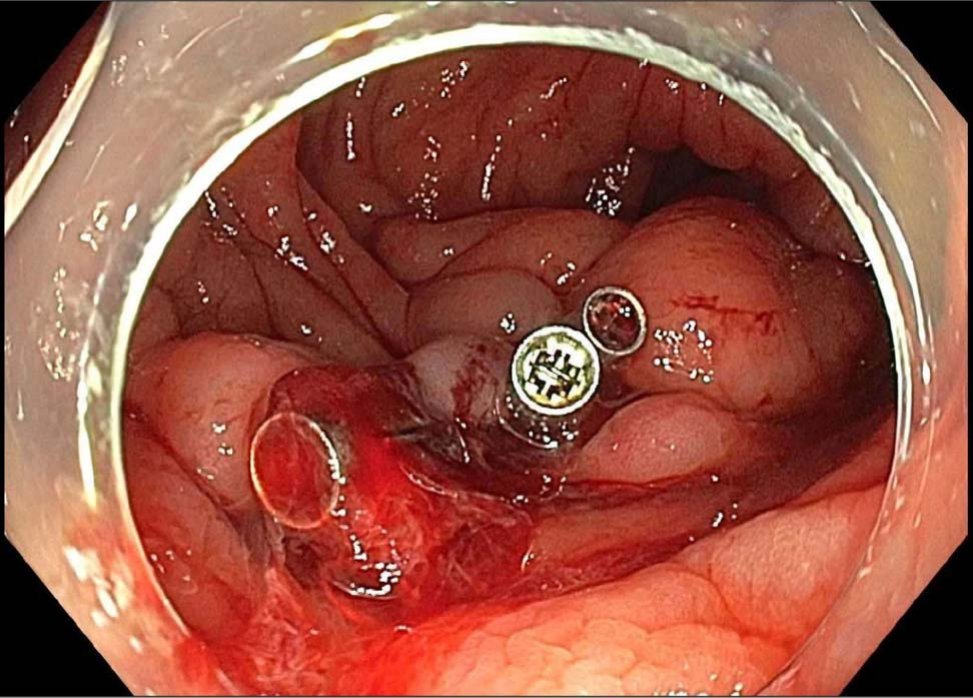
Culprit varix clipped through the scope clip.

**Figure 6.**
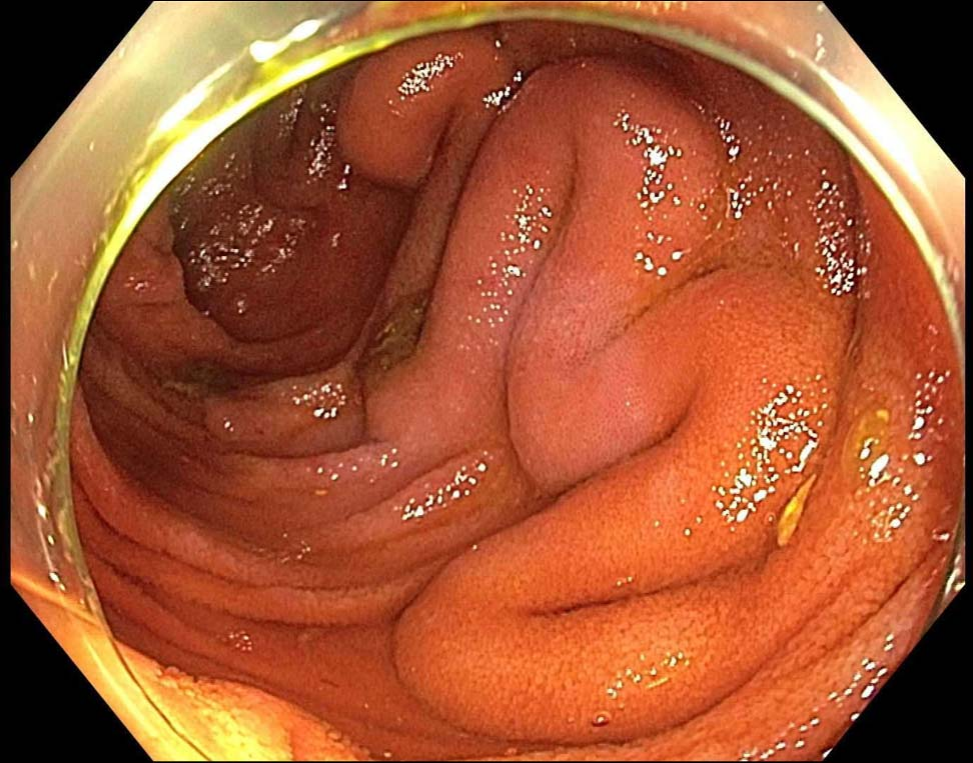
Terminal ileum varices.

He subsequently presented with recurrent hematochezia. Initial hemoglobin was 63 g/L, and urea, C-reactive protein, and liver biochemistry were normal. Repeat abdominal CT reported acute pan-colitis (Figures 1 and 2). He settled with conservative management including blood transfusions and an empirical course of antibiotics for presumed infective colitis. CT angiogram was performed during repeat admission with identical symptoms. It reported “extensive multifocal angiodysplasia” in the distal ileum and entire colon. This suggests involvement of both superior and inferior mesenteric vein territory, once again without evidence of cirrhosis or portal hypertension. FibroScan was normal (median kPa = 4.8, interquartile range 17%, controlled attenuation parameter 218). Inpatient colonoscopy revealed large pan-colonic varices (Figure 3) and terminal ileum varices (Figure 6) with the likely culprit an ascending colon varix with stigmata of recent bleeding (Figure 4). This was treated with the application of through-the-scope clips (Figure 5). Considering other potential endoscopic treatment modalities, band ligation was not feasible due to the proximal colonic location. In addition, our center had no experience in banding colonic varices. Sclerotherapy was also not pursued, in line with international guidelines.^4^ Metoprolol was changed to low-dose carvedilol with gradual dose increment. Subsequent outpatient follow-up found him asymptomatic with normalized hemoglobin and good tolerance to maximal dose of carvedilol. Repeat imaging with a CT scan was not performed as there was no clinical indication.

## DISCUSSION

Bleeding ICV is an exceptionally rare condition with challenging management. There are no standardized treatment protocols, and even case reports are sparse. Although portal hypertension related variceal management is well established, little is known about these idiopathic cases.^1^

The demographic profile of ICV patients varies, most reports indicate association with male gender presenting at a younger age.^5^ Associated iron deficiency anemia and lower gastrointestinal bleeding, ranging from mild and intermittent to severe and life-threatening are common.^6,7^

The ICV pathophysiology remains incompletely understood. Several hypotheses have been proposed, including congenital weakness or malformation of the colonic venous system, localized microscopic venous outflow obstruction, and developmental anomalies of mesenteric venous drainage.^8–14^ Some reports describe familial clustering, suggesting a possible genetic or heritable predisposition.^11,12^ In addition, a subset of cases may reflect a form of segmental idiopathic portal hypertension, wherein localized increases in venous pressure occur in the absence of systemic portal hypertension or liver disease.^15,16^ These theories, although unproven, support the heterogeneity of this rare condition and emphasize the need for thorough exclusion of secondary causes.

The ICV diagnosis is usually established endoscopically or radiologically. Colonoscopy remains the gold standard, allowing for direct assessment and potential therapeutic interventions.^7^ In addition to standard protocol of CT abdomen, angiography by CT or magnetic resonance imaging is valuable in delineating the extent, ruling out other vascular abnormalities, or even identifying the bleeding point.^17,18^ These tests are just as important in ruling out far more common etiologies, especially portal hypertension with or without cirrhosis. However, as illustrated by our case, radiology can lead to a misleading diagnosis such as colitis or angiodysplasia, as some radiologists may not be familiar with this rare entity.

The ICV management depends on the severity of bleeding, departmental experience, and available equipment.^2,5–7^ Conservative approach has been used in cases of mild bleeding.^2,7^ Endoscopic interventions, such as clipping, band ligation, and sclerotherapy, have been used with varying success. Surgical resection is reserved for refractory cases or those with uncontrollable severe bleeding.^2^

There is lack of evidence of pharmacological treatment. NSBBs, such as propranolol or carvedilol, have proven effective in the management of portal hypertension.^3^ However, its use in ICV is uncharted. We used carvedilol as secondary prophylaxis after the initial endoscopic hemostasis in our case. This proved to be both efficient and well tolerated.

We showcase a successful management of both acute bleeding and secondary prophylaxis, thus adding valuable information to the limited body of evidence. It underscores the potential benefit of NSBBs such as carvedilol in treating ICV, extending beyond the realm of portal hypertension.

In conclusion, symptomatic ICV remain diagnostic and therapeutic challenge. Primary endoscopic hemostasis followed by recurrent bleeding risk reduction with carvedilol could be a viable treatment approach.

## DISCLOSURES

Author contributions: A. Ravikulan: Drafted case report, looked through previous notes, literature review, image selection; R. Gearry: Reviewing draft, reviewing images, endoscopist involved in case; J. Kubovy: Drafting, reviewing draft, image selection, endoscopist involved in case. A. Ravikulan is guarantor of the article.

Financial disclosure: None to report.

Informed consent was obtained for this case report.
